# Self‐Application of Aging Stereotypes as an Independent Predictor of Loneliness in Community‐Dwelling Older Adults: A Cross‐Sectional Study

**DOI:** 10.1111/ggi.70822

**Published:** 2026-09-01

**Authors:** Rahime Aslan, Mahsun Toplu

**Affiliations:** ^1^ Faculty of Health Sciences, Department of Nursing, Psychiatric Nursing Division Kütahya Health Sciences University Kütahya Turkey; ^2^ Faculty of Health Sciences, Department of Nursing Kütahya Health Sciences University Kütahya Turkey

**Keywords:** aged, ageism, loneliness, self concept, stereotyping

## Abstract

**Aim:**

Loneliness is common in later life and may be shaped by aging stereotypes. Drawing on Stereotype Embodiment Theory, this study examined whether the components of aging‐stereotype internalization predict loneliness independently of sociodemographic factors and whether effects differ by dimension.

**Methods:**

In this cross‐sectional study, 485 community‐dwelling adults aged 65 or older in Turkey completed the Elderly Internalized Stigma Scale (EISS), indexing awareness, agreement, and self‐application, and the Social and Emotional Loneliness Scale for Adults (SELSA‐S). Spearman's correlations and multiple linear regression were used, with separate models for total, emotional, and social loneliness.

**Results:**

Self‐application showed the strongest bivariate association with loneliness (*ρ* = 0.25, *p* < 0.001), whereas agreement was unrelated. It was independently associated with total loneliness (*β* = 0.23) and both dimensions (*β* = 0.18 emotional, *β* = 0.21 social; all *p* < 0.001), whereas awareness was not. Agreement showed a negative coefficient consistent with suppression. Being unmarried was the strongest correlate of emotional loneliness (*β* = 0.55) but was unrelated to social loneliness (*β* = 0.04), for which self‐application had the largest standardized coefficient among the included predictors. Separating the components explained more variance than the composite score.

**Conclusion:**

Loneliness was associated with applying aging stereotypes to oneself rather than with awareness or general agreement, and this held for both dimensions. Being married was associated with lower emotional loneliness but not with social loneliness. Findings for social loneliness should be interpreted cautiously given the low internal consistency of that subscale. Interventions targeting self‐directed stereotypes merit evaluation.

## Graphical abstract

In 485 community‐dwelling older adults, loneliness was associated with applying aging stereotypes to oneself, not with awareness of them or general agreement. Self‐application was independently associated with both emotional and social loneliness, suggesting that interventions should target the self‐relevant component of internalization rather than awareness alone.

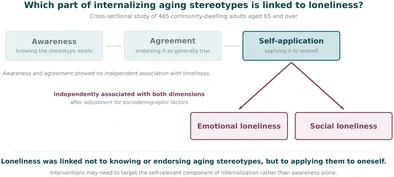

## 1 Introduction

Globally, one in six people experiences loneliness, which contributes to roughly 871 000 deaths each year, and the burden falls disproportionately on older adults [1]. Loneliness is not simply the absence of social contact; it reflects a subjective gap between the relationships a person has and those they desire [2], and social loneliness, an unmet need for a broader network, need not move together with emotional loneliness, the absence of close bonds [2, 3]. In later life it is linked to sex, employment, education, and network size [4, 5, 6], and marital status is among its most robust correlates, with loss of a spouse exerting a particularly pronounced effect [7, 8]. In a Turkish community sample, ageism and loneliness were both negatively associated with successful aging [9]. Most of these risk factors are events that may be difficult to reverse once they have occurred; beliefs about one's own aging differ in this respect, being acquired over the life course, open to psychosocial modification, and specific to old age.

Ageism comprises the stereotypes, prejudice, and discrimination directed at people on the basis of age [10], and since the term was introduced [11] a central insight has been that such attitudes may be turned inward, toward oneself. Stereotype Embodiment Theory specifies how this inward form develops: negative age stereotypes are acquired across the life course before they become personally relevant, can operate without awareness, and are embodied in old age as self‐perceptions of aging that are held to affect health through psychological, behavioral, and physiological pathways [12]. Applied to loneliness, the theory implies a behavioral route in which older adults who apply aging stereotypes to themselves expect rejection, take part in fewer activities, and withdraw from contact, each response widening the gap between the relationships they have and those they want [12, 13].

Testing this requires attention to measurement. The instrument used here distinguishes three sequential components of age‐stereotype processing [14]: awareness, agreement, and self‐application, of which self‐application most directly represents the stage at which aging stereotypes become personally relevant; endorsing a stereotype while excluding oneself from the group it describes is a documented alternative response [15]. Summing the three therefore combines distinct stages and can obscure their separate associations with an outcome. Empirical work is consistent with a self‐relevant route: age‐based devaluation appears to reach loneliness partly through the individual's own aging self‐perceptions [13, 16], and internalized aging stereotypes have been linked to poorer mental health with loneliness as a mediator [17]. Which part of the internalization process carries the association has not been established, and because international loneliness policies have focused mainly on high‐income countries [18], the gap is especially significant in middle‐income settings.

### 1.1 Study Aims

This study aimed to examine the association between the internalization of aging stereotypes and loneliness in community‐dwelling older adults and to determine whether it predicts loneliness independently of sociodemographic factors. Because the loneliness measure distinguishes emotional from social loneliness, each dimension was also modeled separately, and the three stereotype components were examined both separately and as a composite.

## 2 Methods

### 2.1 Study Design and Sampling

This cross‐sectional, correlational study was conducted among adults aged 65 and over living in the city center of Kütahya, Turkey, drawn from the 80 370 residents of Kütahya in that age group recorded by the Turkish Statistical Institute in 2021 [19]. An a priori G*Power analysis for a nine‐predictor multiple regression, assuming *α* = 0.05, power = 0.80, and a small incremental effect (*f* ^2^ = 0.02) for two tested predictors, indicated a required sample of 485 [20]; a sensitivity analysis for the achieved sample is reported in the Supporting Information. Participants were recruited with equal allocation (about 162 per stratum) from three neighborhoods classified by the Kütahya Provincial Governorate as low, middle, or high in socioeconomic level. Volunteers meeting the eligibility criteria were enrolled through communal areas identified by local headmen until each quota was filled; selection within strata was not random, so this is a nonprobability sample. Neighborhood socioeconomic level structured recruitment only; participants' own perceived income adequacy was recorded separately.

### 2.2 Participants

The study included community‐dwelling volunteers aged 65 and over who resided in the city center of Kütahya and were literate in Turkish. Individuals who might have difficulty responding to scale items due to a diagnosis of dementia, legal guardianship, or sensory impairment were excluded.

### 2.3 Data Collection Instruments

Data were collected through face‐to‐face interviews between June 20 and September 30, 2025, with each interview lasting approximately 25–30 min. Three instruments were used:

The Sociodemographic Characteristics Form comprised 13 items covering age, sex, education, marital status, perceived income adequacy (income below, equal to, or above expenses; referred to below as low, middle, and high income), children and grandchildren, cohabitation, employment, experience of age‐based discrimination, chronic illness, frequency of healthcare use, and functional dependence, the last recorded with a single self‐report item on whether help from another person was needed for daily activities.

The Elderly Internalized Stigma Scale (EISS), developed by Karasoy and Baydur [14] from a progressive model of self‐stigma [21], assesses three sequential components of age‐stereotype processing on a 3‐point format: stereotype awareness (5 items), agreement (5 items), and self‐application (7 items). Following the original scoring, items were reverse‐coded and each subscale linearly transformed to a 0–100 metric, with the total taken as the mean of the three subscales; higher scores indicate greater internalization. In the present sample *α* was 0.86 for the total scale, 0.81 for awareness, 0.77 for agreement, and 0.74 for self‐application, against 0.89, 0.82, 0.67, and 0.88 in the development study [14]; the self‐application subscale was thus less consistent here than in the original. Because *α* is downwardly biased for three‐category items, McDonald's *ω* was also computed: 0.92 for the total scale, 0.88 for awareness, 0.78 for agreement, and 0.81 for self‐application.

The Social and Emotional Loneliness Scale for Adults (SELSA‐S), developed by DiTommaso and Spinner [2], follows Weiss's distinction between emotional and social isolation [3]. The 15‐item short form yields a social and an emotional subscale, the latter combining the family and romantic items, which together form the total. Items are rated on a 7‐point scale, with totals ranging from 15 to 105 and higher scores indicating greater loneliness. In the Turkish adaptation, *α* was 0.83 for the total scale, 0.82 for the social subscale, and 0.76 and 0.85 for the family and romantic components of the emotional subscale [22]; in the present sample it was 0.79 for the total scale and 0.80 for the emotional dimension but only 0.49 for the five‐item social dimension, and findings for that dimension are interpreted with the caution set out in the Section 4.4.

### 2.4 Data Analysis

Data were analyzed in IBM SPSS Statistics 26.0, and the factor analyses reported as Supporting Information in JASP 0.19. Bivariate associations were examined with Spearman's rank‐order correlations, and group differences with independent‐samples *t*‐tests (Welch‐corrected where appropriate) and one‐way ANOVA with Games–Howell post hoc tests, reporting Cohen's *d* and *η* ^2^. Internal consistency was estimated with Cronbach's *α* and McDonald's *ω*.

Because the EISS is used here at the subscale level, its structure was examined in the same sample by ordinal confirmatory factor analysis with WLSMV and by exploratory factor analysis with minimum residual extraction and oblique rotation; full results are reported in the Supporting Information and their implications in Section 4.5.

Loneliness was modeled by multiple linear regression for total, emotional, and social loneliness. For each outcome the primary model entered the three EISS components separately, because, per Stereotype Embodiment Theory [12], self‐application most directly reflects internalization; a secondary model entered the composite score alone, for comparability with prior total‐score research, and the two were compared on adjusted *R* ^2^. Covariates were fixed in advance from established correlates of later‐life loneliness, not chosen on bivariate significance: age, sex, marital status, income, employment, and functional dependence [4, 5, 6, 7, 8]. Living arrangement was omitted because it overlaps with marital status; educational attainment, a plausible confounder of both, was entered in a sensitivity analysis rather than the primary model to limit the number of predictors. Categorical predictors were dummy‐coded; because the low‐ and high‐income groups did not differ on SELSA‐S scores (*p* = 0.536), they were merged and used as the reference. Linearity, multicollinearity, influential observations, independence of residuals, normality of residuals, and homoscedasticity were examined; the first four raised no concern, whereas residual variance was uneven and residuals were positively skewed, as noted in Section 4.5. Significance was set at *p* < 0.05.

## 3 Results

### 3.1 Descriptive Statistics

Sociodemographic characteristics are given in Table 1 and descriptive statistics for the EISS and SELSA‐S scores in Table 2. Skewness and kurtosis fell within ±1 [23], but because normality tests flagged minor departures and the items are ordinal, Spearman's coefficients were used.

**TABLE 1:** Comparison of Social and Emotional Loneliness and Elderly Internalized Stigma Scale Mean Scores by sociodemographic characteristics of participants (*N* = 485).

Variable	*n* (%)	SELSA‐S (*M* ± SD)	SELSA‐S test	EISS (*M* ± SD)	EISS test
Age group					
65–74	366 (75.5)	37.42 ± 15.45	*t* = −0.21 *p* = 0.830 *d* = 0.02	63.76 ± 24.12	*t* = −0.68 *p* = 0.490 *d* = 0.07
≥ 75	119 (24.5)	37.82 ± 18.27		65.43 ± 22.78	
Sex					
Male	287 (59.2)	37.17 ± 16.68	*t* = −0.566 *p* = 0.572 *d* = 0.052	63.59 ± 24.90	*t* = −0.651 *p* = 0.515 *d* = 0.059
Female	198 (40.8)	38.02 ± 15.43		65.00 ± 22.10	
Educational attainment					
Primary school	258 (53.2)	39.78 ± 16.66	*F*(2, 482) = 5.573 *p* = 0.004** *η* ^2^ = 0.023	71.66 ± 20.88	*F*(2, 482) = 40.522 *p* < 0.001*** *η* ^2^ = 0.144
Secondary school	145 (29.9)	35.30 ± 16.36		60.30 ± 23.47	
University	82 (16.9)	34.30 ± 12.96		47.44 ± 22.88	
Games–Howell post hoc, SELSA‐S: primary > secondary (*p* = 0.010), primary > university (*p* = 0.002). EISS: primary > secondary (*p* < 0.001), primary > university (*p* < 0.001), secondary > university (*p* < 0.001).					
Income level					
Low	162 (33.4)	35.41 ± 15.22	*F*(2, 482) = 4.796 *p* = 0.009** *η* ^2^ = 0.020	63.45 ± 21.80	*F*(2, 482) = 0.392 *p* = 0.676 *η* ^2^ = 0.002
Middle	161 (33.2)	40.66 ± 16.62		63.53 ± 25.66	
High	162 (33.4)	36.49 ± 16.22		65.52 ± 23.76	
Games–Howell post hoc, SELSA‐S: middle > low (*p* = 0.003), middle > high (*p* = 0.024).					
Marital status					
Married	360 (74.2)	32.98 ± 14.18	*t* = −11.880 *p* < 0.001*** *d* = 1.233	63.13 ± 23.16	*t* = −1.632 *p* = 0.103 *d* = 0.169
Unmarried	125 (25.8)	50.58 ± 14.42		67.16 ± 25.34	
Having children					
Yes	427 (88.0)	37.13 ± 15.93	*t* = −1.402 *p* = 0.161 *d* = 0.196	64.45 ± 23.86	*t* = 0.706 *p* = 0.481 *d* = 0.099
No	58 (12.0)	40.31 ± 17.71		62.09 ± 23.31	
Having grandchildren					
Yes	373 (76.9)	36.69 ± 15.84	*t* = −2.045 *p* = 0.041* *d* = 0.220	65.37 ± 23.19	*t* = 2.041 *p* = 0.042* *d* = 0.220
No	112 (23.1)	40.25 ± 17.01		60.15 ± 25.34	
Living arrangement					
With spouse	264 (54.4)	32.86 ± 14.21	*F*(2, 482) = 57.607 *p* < 0.001*** *η* ^2^ = 0.193	63.53 ± 23.55	*F*(2, 482) = 1.875 *p* = 0.154 *η* ^2^ = 0.008
Living alone	130 (26.8)	36.92 ± 14.19		62.48 ± 23.04	
Other	91 (18.8)	51.87 ± 15.93		68.42 ± 25.10	
Games–Howell post hoc, SELSA‐S: other > with spouse (*p* < 0.001), other > living alone (*p* < 0.001), living alone > with spouse (*p* = 0.023). EISS: no significant pairwise differences.					
Employment status					
Retired/not employed	453 (93.4)	37.02 ± 15.99	*t* = −2.561 *p* = 0.011* *d* = 0.468	64.05 ± 23.82	*t* = −0.398 *p* = 0.691 *d* = 0.073
Employed	32 (6.6)	44.56 ± 17.26		65.79 ± 23.51	
Experience of age‐based discrimination					
Yes	118 (24.3)	37.84 ± 18.90	*t* = 0.224 *p* = 0.823 *d* = 0.027	71.89 ± 20.75	*t* = 4.442 *p* < 0.001*** *d* = 0.435
No	367 (75.7)	37.41 ± 15.21		61.68 ± 24.19	
Functional dependence					
Yes	121 (24.9)	41.93 ± 18.82	*t* = 3.114 *p* = 0.002** *d* = 0.367	74.44 ± 18.93	*t* = 6.372 *p* < 0.001*** *d* = 0.592
No	364 (75.1)	36.05 ± 14.93		60.75 ± 24.28	
Chronic illness					
Yes	287 (59.2)	37.09 ± 16.02	*t* = −0.691 *p* = 0.490 *d* = 0.064	67.38 ± 23.23	*t* = 3.614 *p* < 0.001*** *d* = 0.334
No	198 (40.8)	38.13 ± 16.40		59.52 ± 23.86	
Frequency of healthcare use					
Several times/week	51 (10.5)	35.47 ± 15.57	*F*(2, 482) = 1.243 *p* = 0.290 *η* ^2^ = 0.005	72.72 ± 17.82	*F*(2, 482) = 5.005 *p* = 0.007** *η* ^2^ = 0.020
Several times/month	365 (75.3)	37.33 ± 15.88		63.95 ± 24.21	
Never	69 (14.2)	40.00 ± 17.86		58.99 ± 23.80	
Games–Howell post hoc, EISS: several times/week > several times/month (*p* = 0.003), several times/week > never (*p* < 0.001).					

*Note: N* = 485. Values are *M* ± SD. Two‐group comparisons used independent‐samples *t*‐tests (Welch‐corrected where variances were unequal) with Cohen's *d*; three‐group comparisons used one‐way ANOVA with *η* ^2^ and Games–Howell post hoc tests.

Abbreviations: EISS = Elderly Internalized Stigma Scale; SELSA‐S = Social and Emotional Loneliness Scale for Adults.

* *p* < 0.05.

** *p* < 0.01.

*** *p* < 0.001.

**TABLE 2:** Spearman's rank‐order correlations among EISS and SELSA‐S total and subscale scores (*N* = 485).

Variable	*M*	SD	1	2	3	4	5	6	7
EISS—Stereotype awareness	58.35	33.51	1.00						
EISS—Stereotype agreement	83.13	25.07	0.41**	1.00					
EISS—Stereotype self‐application	51.02	28.95	0.64**	0.35**	1.00				
EISS—Total	64.17	23.83	0.89**	0.61**	0.86**	1.00			
SELSA‐S—Social loneliness	12.99	6.22	0.19**	−0.01	0.24**	0.19**	1.00		
SELSA‐S—Emotional loneliness	24.52	12.83	0.13*	−0.02	0.21**	0.16**	0.28**	1.00	
SELSA‐S—Total	37.51	16.20	0.18**	−0.03	0.25**	0.19**	0.62**	0.90**	1.00

*Note: N* = 485. Values are Spearman's rank‐order correlation coefficients (*ρ*). The Elderly Internalized Stigma Scale (EISS) comprises three subscales (stereotype awareness, stereotype agreement, and stereotype self‐application) and a total score (0–100). The SELSA‐S yields social and emotional loneliness subscales and a total score. Correlations between each total and its own subscales are part–whole associations and are reported for completeness.

* *p* < 0.01.

** *p* < 0.001.

### 3.2 Comparison of Loneliness and Internalized Aging Stereotypes by Sociodemographic Characteristics

Mean SELSA‐S and EISS scores by sociodemographic characteristics are presented in Table 1; loneliness varied most with marital status, unmarried participants scoring substantially higher than married ones (*d* = 1.23, the largest effect in the study), and with living arrangement (*η* ^2^ = 0.19), whereas internalized stereotypes differed by neither and varied most with education (*η* ^2^ = 0.14). Age was unrelated to both outcomes, whether treated as continuous (*ρ* = −0.05 and 0.06) or as two groups (65–74, *n* = 366, versus 75 or older, *n* = 119; all *p* > 0.05).

### 3.3 Relationships Among the EISS and SELSA‐S Dimensions

The three stereotype dimensions related to loneliness in clearly different ways (Table 2). Self‐application showed the strongest associations, with the SELSA‐S total (*ρ* = 0.25) and its social (*ρ* = 0.24) and emotional (*ρ* = 0.21) subscales, all *p* < 0.001; awareness was more weakly related (*ρ* = 0.18, *p* < 0.001), and agreement was unrelated bivariately (*ρ* = −0.03).

### 3.4 Subscale‐Level Predictors of Total, Emotional, and Social Loneliness: Multiple Linear Regression

Regression results are given in Tables 3, 4, 5. All three models were significant, *F*(9, 475) = 28.32, 37.50, and 6.32, respectively, all *p* < 0.001, but differed considerably in explanatory power, accounting for 33.7%, 40.4%, and 9.0% of the variance (adjusted *R* ^2^). For each outcome the corresponding model using the EISS total score explained less (adjusted *R* ^2^ = 0.287, 0.369, and 0.056), so separating the composite improved prediction throughout. Adding educational attainment as a covariate changed nothing of substance: no education term was significant and self‐application remained a significant predictor of all three outcomes.

**TABLE 3:** Multiple linear regression predicting total loneliness from sociodemographic covariates, age, and EISS subscales (*N* = 485).

Variable	*B*	SE	*β*	*t*	*p*	95% CI
Age	−0.152	0.107	−0.054	−1.416	0.157	[−0.363, 0.059]
Sex (ref: female)	3.518	1.291	0.107	2.725	0.007**	[0.981, 6.055]
Income level (ref: low or high income)	4.148	1.334	0.121	3.109	0.002**	[1.527, 6.769]
Marital status (ref: married)	16.797	1.426	0.454	11.775	< 0.001***	[13.994, 19.599]
Employment status (ref: not employed)	6.541	2.438	0.100	2.683	0.008**	[1.750, 11.332]
Functional dependence (ref: no)	3.806	1.462	0.102	2.603	0.010*	[0.933, 6.679]
EISS—Stereotype awareness	0.043	0.024	0.089	1.778	0.076	[−0.005, 0.091]
EISS—Stereotype agreement	−0.119	0.027	−0.184	−4.419	< 0.001***	[−0.172, −0.066]
EISS—Stereotype self‐application	0.126	0.028	0.226	4.511	< 0.001***	[0.071, 0.181]
*R* ^2^ = 0.349, adjusted *R* ^2^ = 0.337, *F*(9, 475) = 28.32, *p* < 0.001						

*Note: N* = 485. Dependent variable: total loneliness (SELSA‐S). VIF values ranged from 1.02 to 1.84, indicating no multicollinearity, and no observation exceeded a Cook's distance of 1. For comparison, the composite model (same covariates + EISS total score instead of subscales) yielded Adjusted *R* ^2^ = 0.287 with EISS total *β* = 0.13 (*p* = 0.001); decomposing the EISS into subscales increased explained variance and revealed divergent subscale effects (self‐application positive, agreement negative, awareness nonsignificant).

Abbreviations: *B*: unstandardized coefficient; CI: confidence interval; SE: standard error; *β*: standardized coefficient.

* *p* < 0.05.

** *p* < 0.01.

*** *p* < 0.001.

**TABLE 4:** Multiple linear regression predicting emotional loneliness from sociodemographic covariates, age, and EISS subscales (*N* = 485).

Variable	*B*	SE	*β*	*t*	*p*	95% CI
Age	−0.119	0.081	−0.053	−1.481	0.139	[−0.278, 0.039]
Sex (ref: female)	2.293	0.969	0.088	2.366	0.018*	[0.389, 4.198]
Income level (ref: low or high income)	3.484	1.001	0.128	3.479	0.001**	[1.516, 5.452]
Marital status (ref: married)	16.246	1.071	0.554	15.170	< 0.001***	[14.141, 18.350]
Employment status (ref: not employed)	3.426	1.831	0.066	1.872	0.062	[−0.170, 7.023]
Functional dependence (ref: no)	2.626	1.098	0.089	2.392	0.017*	[0.469, 4.783]
EISS—Stereotype awareness	0.026	0.018	0.069	1.443	0.150	[−0.010, 0.062]
EISS—Stereotype agreement	−0.084	0.020	−0.165	−4.164	< 0.001***	[−0.124, −0.045]
EISS—Stereotype self‐application	0.081	0.021	0.183	3.864	< 0.001***	[0.040, 0.122]
*R* ^2^ = 0.415, Adjusted *R* ^2^ = 0.404, *F*(9, 475) = 37.50, *p* < 0.001						

*Note: N* = 485. Dependent variable: emotional loneliness (SELSA‐S). The same predictors were entered as in Table 3. VIF values ranged from 1.02 to 1.84, indicating no multicollinearity, and no observation exceeded a Cook's distance of 1. For comparison, the composite model (same covariates + EISS total score instead of subscales) yielded Adjusted *R* ^2^ = 0.369 with EISS total *β* = 0.09 (*p* = 0.014).

Abbreviations: *B*: unstandardized coefficient; CI: confidence interval; SE: standard error; *β*: standardized coefficient.

* *p* < 0.05.

** *p* < 0.01.

*** *p* < 0.001.

**TABLE 5:** Multiple linear regression predicting social loneliness from sociodemographic covariates, age, and EISS subscales (*N* = 485).

Variable	*B*	SE	*β*	*t*	*p*	95% CI
Age	−0.033	0.048	−0.030	−0.677	0.499	[−0.127, 0.062]
Sex (ref: female)	1.225	0.580	0.097	2.111	0.035*	[0.085, 2.365]
Income level (ref: low or high income)	0.663	0.599	0.050	1.107	0.269	[−0.514, 1.841]
Marital status (ref: married)	0.551	0.641	0.039	0.859	0.391	[−0.709, 1.810]
Employment status (ref: not employed)	3.114	1.096	0.125	2.843	0.005**	[0.962, 5.267]
Functional dependence (ref: no)	1.180	0.657	0.082	1.796	0.073	[−0.111, 2.471]
EISS—Stereotype awareness	0.017	0.011	0.091	1.547	0.123	[−0.005, 0.038]
EISS—Stereotype agreement	−0.035	0.012	−0.141	−2.876	0.004**	[−0.059, −0.011]
EISS—Stereotype self‐application	0.045	0.013	0.210	3.583	< 0.001***	[0.020, 0.070]
*R* ^2^ = 0.107, adjusted *R* ^2^ = 0.090, *F*(9, 475) = 6.32, *p* < 0.001						

*Note: N* = 485. Dependent variable: social loneliness (SELSA‐S). The same predictors were entered as in Table 3. VIF values ranged from 1.02 to 1.84, indicating no multicollinearity, and no observation exceeded a Cook's distance of 1. For comparison, the composite model (same covariates + EISS total score instead of subscales) yielded adjusted *R* ^2^ = 0.056 with EISS total *β* = 0.15 (*p* = 0.001).

Abbreviations: *B*: unstandardized coefficient; CI: confidence interval; SE: standard error; *β*: standardized coefficient.

* *p* < 0.05.

** *p* < 0.01.

*** *p* < 0.001.

The EISS effects were consistent across outcomes. Self‐application independently predicted total loneliness (*β* = 0.23) and both dimensions (*β* = 0.18 emotional, *β* = 0.21 social), all *p* < 0.001, whereas awareness predicted none (*p* = 0.076, 0.150, and 0.123). Agreement was a significant negative predictor in all three models (*β* = −0.18, −0.17, and −0.14) once the other subscales were controlled; because it was unrelated bivariately, this is best read as statistical suppression, discussed below.

The sociodemographic predictors behaved differently across the two dimensions. Marital status was the strongest predictor of total loneliness (unmarried *β* = 0.45) and dominated the emotional dimension (*β* = 0.55, both *p* < 0.001) but was unrelated to social loneliness (*β* = 0.04, *p* = 0.391), whereas employment showed the reverse pattern, predicting social loneliness (*β* = 0.13, *p* = 0.005) but not the emotional dimension (*β* = 0.07, *p* = 0.062). Male sex predicted all three outcomes, and income and functional dependence the total and emotional models; age predicted none. Within the social loneliness model, self‐application was the strongest predictor of any variable entered.

## 4 Discussion

This study examined how internalized aging stereotypes relate to loneliness in community‐dwelling older adults. When the construct was separated into its components, the association was carried by self‐application alone, and modeling the two loneliness dimensions separately showed that this holds for both, whereas the sociodemographic predictors are dimension‐specific.

### 4.1 Stereotype Self‐Application as the Active Internalization Mechanism

Self‐application predicted loneliness independently of sociodemographic factors and age, whereas awareness did not and agreement did not at the bivariate level. This is what Stereotype Embodiment Theory predicts: an older adult may recognize aging stereotypes, and even regard them as broadly true, without reporting greater loneliness; higher loneliness was observed primarily when they were applied to the self [12, 17]. The pattern also explains why the composite score performed less well in every outcome, since summing the three components dilutes the contribution of self‐application with two that do not share its relationship to loneliness.

The finding specifies the route described by existing work on ageism and loneliness [13, 16]. What accounts for the association is not exposure to stereotypes, nor general endorsement of them, but their application to oneself.

The negative coefficient for agreement warrants caution. Agreement was unrelated to loneliness on its own and turned negative only when self‐application and awareness were controlled, which points to statistical suppression rather than a protective effect. One possible reading is that older adults who regard aging stereotypes as broadly true but decline to apply them to themselves are distancing themselves from the stereotyped group rather than internalizing it [15], and the coefficient would be consistent with such dissociation. The recurrence of the coefficient across the loneliness outcomes indicates that it is not confined to a single scoring specification; because those outcomes are correlated and partly overlapping, however, this does not constitute independent replication. Dissociation was not measured in this study, so the reading remains a hypothesis requiring direct assessment.

### 4.2 Differentiated Effects on Emotional and Social Loneliness

Self‐application was associated with both dimensions and agreement retained its negative coefficient in both, but the models differed elsewhere. Marital status dominated emotional loneliness, of which the model explained 40%, whereas it did not predict social loneliness at all; there the model explained 9% and self‐application was the strongest predictor entered. The contrast partly follows from what the dimensions measure: emotional loneliness reflects the absence of a close attachment figure [3] and marital status lies close to that definition. It may also reflect item‐content overlap, since the emotional score combines family and romantic items and the married–unmarried difference was far larger on the romantic component, though it remained moderate on the family component (Table S7). Social loneliness, by contrast, was related to self‐application and to continued employment rather than to partnership, consistent with the behavioral route specified by Stereotype Embodiment Theory [12, 13]. Because the social model explained far less variance and its outcome had limited internal consistency (see Section 4.5), these results support the conclusion that self‐application was associated with both dimensions, not that it was more strongly associated with one.

### 4.3 Sociodemographic Correlates

Marital status was the strongest sociodemographic correlate of loneliness, consistent with systematic‐review [4] and cross‐national evidence [24], and the dimension‐specific analysis places that association in emotional loneliness; living arrangement followed the same logic, indicating that the presence of others does not compensate for the absence of an intimate bond [2, 25]. Male sex was associated with higher loneliness on both dimensions in the adjusted models, consistent with norms discouraging male disclosure [26, 27], education was inversely related to both outcomes and most strongly to stereotypes [12, 28, 29], and functional dependence predicted both [30, 31]. The remaining associations, involving employment, income [32], chronic illness [33], and having grandchildren, were outcome‐specific, rested in some cases on small groups, and were unexpected in direction; they were not central to the study hypotheses and require replication.

### 4.4 Clinical and Policy Implications

The findings suggest that interventions should address the application of aging stereotypes to the self rather than awareness alone, an approach on which programs based on Stereotype Embodiment Theory already draw [34, 35]. Because some older adults already decline to apply stereotypes they otherwise regard as true, such programs may build on a response that arises spontaneously rather than having to create it [15]. Such work may complement programs targeting the social determinants of loneliness. Because the study was cross‐sectional, intervention effects require evaluation in longitudinal or experimental research.

### 4.5 Limitations

The cross‐sectional design precludes causal inference, so the direction of the relationship between self‐application and loneliness requires longitudinal study [36]. Measurement warrants particular caution. This is the first large‐scale independent application of the EISS, and although the confirmatory model showed acceptable fit, the exploratory analysis indicated instability and cross‐loadings within the self‐application subscale; the two analyses were run in the same sample, so the confirmatory findings are an internal examination of the proposed structure rather than an independent validation. The three components were strongly intercorrelated and should be read as related stages of one process rather than as independent constructs, and measurement invariance across older‐adult subgroups remains to be established. Imprecision of this kind would generally attenuate rather than inflate the associations reported. The social subscale of the SELSA‐S also showed low internal consistency, which may partly account for the smaller variance explained in that model, so those findings need replication with a more reliable measure. The negative agreement coefficient, interpreted here as suppression, requires independent replication with a direct measure of stereotype dissociation [15]. Functional dependence was indexed by a single self‐report item and reflects perceived need for assistance rather than measured ability. Residuals were positively skewed and heteroscedastic, so the standard errors and confidence intervals in Tables 3, 4, 5 should be read as approximate. Sampling was not probability‐based: the sample was limited to literate, urban older adults and quotas were filled by volunteers approached in communal areas, so those who seldom leave home are probably underrepresented. Finally, the models left substantial variance unexplained.

## 5 Conclusion

In this sample of community‐dwelling older adults, self‐application was, among the three components of aging‐stereotype processing, the one most consistently associated with loneliness: it was independently associated with emotional and social loneliness alike after adjustment for sociodemographic factors and age, whereas neither awareness nor general agreement was. Marital status was the strongest correlate of emotional loneliness but was unrelated to the social dimension, for which self‐application had the largest standardized coefficient among the included predictors. Loneliness in later life therefore appears to depend less on exposure to aging stereotypes than on the degree to which older adults apply them to themselves. Psychosocial interventions addressing self‐application, alongside the social determinants of loneliness, may be worth developing and evaluating, and longitudinal, psychometrically rigorous studies are needed to confirm these effects and establish the measurement model.

## Author Contributions

**Rahime Aslan:** conceptualization, methodology, data collection, formal analysis, investigation, writing – original draft, writing – review and editing, supervision, project administration. **Mahsun Toplu:** data collection, methodology, investigation, writing – review and editing.

## Funding

This research was supported by the Scientific and Technological Research Council of Türkiye (TÜBİTAK) under the University Students Research Projects Support Program (2209‐A; Grant No: 1919B012464103).

## Ethics Statement

This study was approved by the Non‐Interventional Clinical Research Ethics Committee of Kütahya Health Sciences University (Decision No: 2025/06‐47; Date: May 6, 2025). Institutional permission was additionally obtained from the Kütahya Provincial Governorship (Date: June 2, 2025). All procedures were conducted in accordance with the ethical standards of the Declaration of Helsinki.

## Consent

Written informed consent was obtained from all individual participants included in the study prior to data collection.

## Conflicts of Interest

The authors declare no conflicts of interest.

## Supporting information


**Table S1:** Standardized factor loadings for the three‐factor EISS model (WLSMV, *N* = 485).
**Table S2:** Correlations among the three EISS factors (WLSMV, *N* = 485).
**Table S3:** Fit of the three‐factor EISS model (WLSMV, *N* = 485).
**Table S4:** Reliability of the EISS subscales (*N* = 485).
**Table S5:** Pattern matrix for the three‐factor EISS solution (minimum residual extraction, promax rotation, *N* = 485).
**Table S6:** Correlations among the three factors of the exploratory solution (*N* = 485).
**Table S7:** SELSA‐S component scores by marital status (*N* = 485).

## Data Availability

The data that support the findings of this study are available on request from the corresponding author. The data are not publicly available due to privacy or ethical restrictions.
