# Supplementary material for: Self‐Application of Aging Stereotypes as an Independent Predictor of Loneliness in Community‐Dwelling Older Adults: A Cross‐Sectional Study
Source: Geriatr Gerontol Int. 2026 Sep 1;26(9):e70822. doi: 10.1111/ggi.70822 (PMC13534872; doi:10.1111/ggi.70822)
Supplement: Supplementary file 1 [file GGI-26-0-s001.docx]

**Supplementary Material**

*Self-Application of Aging Stereotypes as an Independent Predictor of Loneliness in Community-Dwelling Older Adults: A Cross-Sectional Study*

Manuscript ID 6316647 · Geriatrics & Gerontology International

**Confirmatory Factor Analysis of the Elderly Internalized Stigma Scale (EISS)**

Because the EISS was used at the subscale level in the regression models, its three-factor structure was examined before the main analyses (*N* = 485). The items have three ordered response categories, for which normal-theory estimation is not appropriate; the model was therefore estimated with the robust diagonally weighted least-squares estimator implemented in JASP 0.19 for ordinal indicators (WLSMV), applied to the polychoric correlation matrix, with the items declared ordinal and factor variances fixed at 1. The three factors were allowed to correlate. The confirmatory and exploratory analyses reported here were run on the same sample, which was not split; they therefore constitute an internal examination of the scale’s structure rather than an independent validation of it.

**Table S1**

*Standardized Factor Loadings for the Three-Factor EISS Model (WLSMV, N = 485)*

| **Item** | **λ** | ***SE*** | ***z*** | ***p*** | **95% CI** |
| --- | --- | --- | --- | --- | --- |
| **Stereotype awareness** |  |  |  |  |  |
| Item 1 | .767 | .041 | 18.87 | < .001 | [.688, .847] |
| Item 2 | .794 | .032 | 24.80 | < .001 | [.732, .857] |
| Item 3 | .788 | .030 | 26.39 | < .001 | [.730, .847] |
| Item 4 | .886 | .023 | 38.08 | < .001 | [.840, .932] |
| Item 5 | .926 | .025 | 37.30 | < .001 | [.877, .974] |
| **Stereotype agreement** |  |  |  |  |  |
| Item 6 | .772 | .056 | 13.77 | < .001 | [.662, .882] |
| Item 7 | .767 | .044 | 17.26 | < .001 | [.680, .854] |
| Item 8 | .922 | .038 | 24.48 | < .001 | [.848, .996] |
| Item 9 | .682 | .047 | 14.41 | < .001 | [.590, .775] |
| Item 10 | .866 | .043 | 20.33 | < .001 | [.782, .949] |
| **Stereotype self-application** |  |  |  |  |  |
| Item 11 | .623 | .045 | 13.94 | < .001 | [.535, .710] |
| Item 12 | .716 | .042 | 17.22 | < .001 | [.634, .797] |
| Item 13 | .754 | .036 | 21.23 | < .001 | [.684, .824] |
| Item 14 | .781 | .044 | 17.90 | < .001 | [.695, .866] |
| Item 15 | .619 | .050 | 12.47 | < .001 | [.521, .716] |
| Item 16 | .691 | .041 | 16.99 | < .001 | [.611, .770] |
| Item 17 | .663 | .044 | 15.04 | < .001 | [.576, .749] |

*Note.* *N* = 485. λ: standardized factor loading. Factor variances were fixed at 1, so the loadings are fully standardized. Standard errors are robust.

**Table S2**

*Correlations Among the Three EISS Factors (WLSMV, N = 485)*

| **Factor pair** | **Estimate** | ***SE*** | ***z*** | **95% CI** |
| --- | --- | --- | --- | --- |
| Awareness – Agreement | .604 | .049 | 12.43 | [.509, .699] |
| Awareness – Self-application | .783 | .031 | 25.07 | [.722, .845] |
| Agreement – Self-application | .602 | .047 | 12.81 | [.510, .694] |

*Note.* *N* = 485. All correlations *p* < .001.

**Table S3**

*Fit of the Three-Factor EISS Model (WLSMV, N = 485)*

| **Index** | **Value** | **Conventional criterion** |
| --- | --- | --- |
| χ² (*df* = 116) | 442.50, *p* < .001 | — |
| CFI | .937 | ≥ .90 acceptable |
| TLI | .926 | ≥ .90 acceptable |
| RMSEA [90% CI] | .076 [.069, .084] | ≤ .08 acceptable |
| SRMR | .093 | ≤ .08 acceptable |

Note. *N* = 485. Baseline (independence) model χ²(136) = 5323.90. The χ² statistic and the fit indices are scaled for categorical data. Because χ² is sensitive to sample size, the approximate fit indices are given greater weight. Conventional criteria follow Hu and Bentler (Hu LT, Bentler PM. Struct Equ Model. 1999;6(1):1-55). SRMR (.093) is marginally above the conventional .08 criterion; the remaining indices meet their criteria.

**Table S4**

*Reliability of the EISS Subscales (N = 485)*

| **Subscale** | **Items** | **Cronbach’s α** | **McDonald’s ω** |
| --- | --- | --- | --- |
| Stereotype awareness | 5 | .807 | .884 |
| Stereotype agreement | 5 | .766 | .780 |
| Stereotype self-application | 7 | .737 | .810 |
| Total scale | 17 | .863 | .918 |

*Note.* *N* = 485. ω was derived from the WLSMV measurement model and is the more appropriate coefficient for items with three ordered response categories.

**Interpretation of the Confirmatory Model**

The CFA provided generally acceptable support for the proposed three-factor structure, although the fit was not uniformly strong. All standardized loadings were substantial and significant, ranging from .62 to .93, and the approximate fit indices met conventional criteria with the exception of SRMR, which fell marginally above the .08 threshold. Two qualifications should be noted. First, the self-application items carried the lowest loadings in the model (.62 to .78), so this subscale is measured somewhat less precisely than the other two; such measurement imprecision would generally be expected to attenuate rather than inflate its association with loneliness. Second, the factors were substantially intercorrelated, particularly awareness and self-application (*r* = .78), which indicates that the components are related stages of a single process and not independent constructs; this is consistent with the sequential model on which the scale was built and does not undermine their separate use as predictors, since collinearity in the regression models remained low (all VIF ≤ 1.84). An exploratory solution reported below qualifies this picture at the item level, and both sets of results are addressed in the Limitations section of the main text.

**Exploratory Factor Analysis of the Elderly Internalized Stigma Scale (EISS)**

The same items were also examined without imposing the measurement model. The 17 EISS items were submitted to an exploratory factor analysis in JASP 0.19 with minimum residual extraction and promax rotation, computed from the Pearson correlation matrix; minimum residual extraction does not assume multivariate normality, which Mardia’s test indicated was not tenable (skewness χ²(969) = 3663.28, *p* < .001; kurtosis *z* = 12.71, *p* < .001). Sampling adequacy was satisfactory (KMO = .86, item-level MSA .76 to .93) and Bartlett’s test of sphericity was significant, χ²(136) = 2675.53, *p* < .001. Retention criteria did not fully converge. Parallel analysis based on principal components supported three factors: the third observed eigenvalue (1.250) exceeded its simulated counterpart (1.214), whereas the fourth (1.114) did not (1.169). The Kaiser rule pointed to four factors and factor-analytic parallel analysis, which compares each observed eigenvalue with the mean of the simulated distribution, to five; the fourth and fifth factors under that criterion exceeded their simulated values by 0.196 and 0.001 respectively and had no interpretable item content. The three-factor solution was therefore retained, consistent with component-based parallel analysis, with the sequential model on which the scale was built, and with the confirmatory model reported above. After rotation it accounted for a cumulative 41.3% of the variance (18.6%, 14.3%, and 8.4% for the three factors).

**Table S5**

*Pattern Matrix for the Three-Factor EISS Solution (Minimum Residual Extraction, Promax Rotation, N = 485)*

| **Item** | **Factor 1** | **Factor 2** | **Factor 3** | **Uniqueness** |
| --- | --- | --- | --- | --- |
| **Stereotype awareness (items 1–5)** |  |  |  |  |
| Item 1 | **.442** | .250 | −.036 | .638 |
| Item 2 | **.572** | .124 | −.005 | .578 |
| Item 3 | **.621** | .064 | −.017 | .577 |
| Item 4 | **.937** | −.184 | −.125 | .401 |
| Item 5 | **.948** | −.250 | −.062 | .374 |
| **Stereotype agreement (items 6–10)** |  |  |  |  |
| Item 6 | −.152 | **.652** | .085 | .634 |
| Item 7 | .000 | **.652** | −.037 | .592 |
| Item 8 | −.127 | **.829** | .005 | .419 |
| Item 9 | .044 | **.506** | −.053 | .737 |
| Item 10 | .060 | **.651** | −.073 | .563 |
| **Stereotype self-application (items 11–17)** |  |  |  |  |
| Item 11 | −.062 | −.077 | **.759** | .511 |
| Item 12 | .240 | .282 | .133 | .698 |
| Item 13 | −.111 | .120 | **.787** | .404 |
| Item 14 | **.351** | −.164 | **.450** | .579 |
| Item 15 | **.358** | .161 | −.020 | .788 |
| Item 16 | **.407** | .028 | .154 | .716 |
| Item 17 | **.361** | .110 | .077 | .764 |

*Note. N = 485. Values are pattern coefficients from a minimum-residual extraction with promax rotation. Coefficients of |.30| or above are shown in bold. Items are grouped by the subscale to which they are assigned in the original scale.* Uniqueness is the proportion of item variance not accounted for by the three factors. Item numbering follows the original scale.

**Table S6**

*Correlations Among the Three Factors of the Exploratory Solution (N = 485)*

|  | **Factor 1** | **Factor 2** | **Factor 3** |
| --- | --- | --- | --- |
| Factor 1 | 1.000 | .589 | .613 |
| Factor 2 | .589 | 1.000 | .383 |
| Factor 3 | .613 | .383 | 1.000 |

*Note. N = 485. Factor 1 comprises the stereotype-awareness items, Factor 2 the stereotype-agreement items, and Factor 3 items 11, 13, and 14 of the stereotype self-application subscale. Correlations are between rotated factors (promax).*

**Interpretation of the Exploratory Solution**

The awareness and agreement subscales were reproduced without exception: items 1 to 5 defined the first factor and items 6 to 10 the second, and no item in either set had a secondary coefficient of .30 or above. The self-application subscale behaved differently. Only items 11, 13, and 14 defined the third factor, and item 14 loaded on the awareness factor as well; of the remaining four, items 15, 16, and 17 loaded on awareness alone (.36 to .41) and item 12 reached .30 on no factor. The four items that departed from their intended subscale were also the least well accounted for by the solution, with uniqueness values between .70 and .79. The factors were substantially intercorrelated, most strongly Factors 1 and 3 (*r* = .61). The confirmatory model gave a still higher correlation between the awareness and self-application factors (.78), although the two estimates rest on different item sets, since the exploratory third factor comprises only three of the seven self-application items. Both point to substantial overlap and are consistent with a sequential process in which the three components are stages rather than independent constructs.

Two considerations bear on how far this qualifies the findings reported in the main text. First, the association with loneliness was carried by the three items that most clearly defined the self-application factor. A score formed from items 11, 13, and 14 alone related to total loneliness as strongly as the full seven-item subscale (ρ = .26 and .25 respectively) and more strongly to emotional loneliness (ρ = .24 and .21), whereas the four items that loaded with awareness related to loneliness at almost exactly the level observed for the awareness subscale itself (ρ = .18, .19, and .14 for total, social, and emotional loneliness, against .18, .19, and .13). All these coefficients were significant at *p* ≤ .003. Second, measurement imprecision of this kind would generally be expected to attenuate observed associations, although the consequences of item-level cross-loadings cannot be determined with certainty. A revised item set and replication in an independent sample nevertheless remain necessary, and this is stated in the Limitations section of the main text.

**Item-Content Overlap Between Marital Status and Emotional Loneliness**

The SELSA-S emotional subscale combines family and romantic items. Because unmarried participants are by definition without a partner, part of the association between marital status and emotional loneliness may reflect item content rather than a substantive difference in attachment. The two components were therefore compared separately.

**Table S7**

*SELSA-S Component Scores by Marital Status (N = 485)*

| **Component** | **Married** | **Unmarried** | ***t*** | ***d*** |
| --- | --- | --- | --- | --- |
| Emotional – romantic | 11.12 (6.72) | 23.58 (4.94) | 22.01*** | 1.97 |
| Emotional – family | 9.07 (5.24) | 13.42 (7.78) | 5.81*** | 0.73 |
| Social | 12.79 (5.98) | 13.58 (6.84) | 1.15 | 0.13 |

*Note.* *N* = 485 (married 360, unmarried 125). Values are sums of the five constituent items, *M* (*SD*). Item numbering follows the Turkish adaptation: romantic 3, 6, 10, 14, 15; family 1, 4, 8, 11, 12; social 2, 5, 7, 9, 13. Group comparisons are Welch-corrected independent-samples *t* tests with Cohen’s *d*. *** *p* < .001.

The difference between married and unmarried participants was far larger for the romantic component (*d* = 1.97) than for the family component (*d* = 0.73), and was negligible for social loneliness (*d* = 0.13). Part of the large marital-status coefficient in the emotional loneliness model therefore reflects item-content overlap. The difference on the family component nevertheless remained moderate and significant, so the association is not reducible to that overlap.

**Power Sensitivity Analysis**

The target sample size reported in the main text was derived from an a priori analysis for two tested predictors. Because the models reported here include nine predictors, a sensitivity analysis was also conducted for the achieved sample. At *N* = 485 and α = .05, the design provides 80% power to detect *f*² values of .016 for the unique contribution of a single tested predictor and .033 for the model as a whole, both within the conventional small-effect range.
